# Mepolizumab as a Possible Treatment for Eosinophilic Ureteritis

**DOI:** 10.7759/cureus.106338

**Published:** 2026-04-02

**Authors:** Hridhay Sheth, Kimberly Waggener, Christian Manganti, Rosetta Campbell, Lorie Fleck

**Affiliations:** 1 Department of Urology, University of South Alabama College of Medicine, Mobile, USA; 2 Department of Urology, Wake Forest University, Winston-Salem, USA; 3 Department of Pathology and Laboratory Medicine, University of South Alabama College of Medicine, Mobile, USA

**Keywords:** eosinophilic cystitis, eosinophilic infiltration, eosinophilic ureteritis, flank pain, il-5, mepolizumab

## Abstract

Eosinophilic ureteritis (EU) is a rare inflammatory condition characterized by eosinophilic infiltration of the ureter, often presenting with flank pain, hematuria, or obstructive uropathy. Fewer than 30 cases have been reported in the literature, and no standardized treatment strategy currently exists.

We report the case of a 54-year-old woman with a history of eosinophilic cystitis who developed progressive left-sided flank pain and hydronephrosis following radical cystectomy. Ureteroscopy with biopsy confirmed EU. A six-week course of corticosteroids resulted in partial radiographic improvement but persistent eosinophilic infiltration on repeat biopsy. Given ongoing biopsy-proven eosinophilic inflammation despite corticosteroid therapy, a multidisciplinary discussion between urology and rheumatology led to initiation of mepolizumab (300 mg administered as three 100-mg subcutaneous injections every four weeks) to target IL-5-mediated eosinophilic inflammation.

Following initiation of therapy, the patient experienced progressive clinical improvement, with symptom relief beginning approximately three months after treatment initiation. Repeat imaging demonstrated decreased ureteral inflammation, and follow-up ureteral biopsy showed complete absence of eosinophils, consistent with remission of active disease. The patient has remained clinically stable for approximately 18 months of follow-up without the need for ureteral stenting or additional surgical intervention.

This case suggests a potential therapeutic role for IL-5 pathway inhibition with mepolizumab in EU and highlights the biologic rationale for targeting eosinophil-mediated inflammation in this condition. However, because this report describes a single patient and off-label biologic therapy, these findings should be interpreted cautiously. Further investigation through additional case series, prospective studies, and rare-disease registries will be necessary to better define the role of IL-5-targeted therapies in EU, with potential for future clinical trials if sufficient evidence emerges.

## Introduction

Eosinophilic ureteritis (EU) is a rare condition characterized by eosinophilic inflammation of the ureter, with fewer than 30 cases reported in the literature. It typically presents with symptoms such as increased voiding frequency, dysuria, and hematuria. It may lead to complications, including urinary tract infections, kidney damage, and sepsis [1]. Eosinophils infiltrate the ureteral wall from the submucosa to the adventitia. EU can cause ureteral obstruction due to mural involvement, but does not typically form an intraluminal mass [1]. Because its clinical presentation can mimic more common causes of ureteral obstruction, such as ureteral strictures, malignancy, or urolithiasis, diagnosis often requires histopathologic confirmation.

The exact cause of EU remains unclear and likely varies between cases [2,3]. Proposed triggers include allergens, medications, infections, trauma to the urothelium, or autoimmune reactions [2]. These triggers initiate an acute inflammatory response characterized by eosinophil recruitment and activation, which may transition into a chronic phase marked by persistent eosinophilic infiltration. EU has also been reported in association with other eosinophilic conditions, including eosinophilic gastrointestinal disorders and hypereosinophilic syndromes (HESs), and some patients present with multiple eosinophilic inflammatory processes [4].

Treatment options include corticosteroids to reduce inflammation, antibiotics to address possible colonizing organisms, and anti-inflammatory agents to manage symptoms [5]. Treatment is generally indicated in cases of persistent pain, voiding dysfunction, or ureteral obstruction. In select cases, corticosteroids alone have led to resolution without surgical intervention [5]. However, in more severe or obstructive presentations, surgical resection of the affected ureteral segment followed by end-to-end anastomosis is often required [1-3].

Despite a variety of reported treatment approaches, no standardized or evidence-based management protocol currently exists due to the rarity of the disease. Interleukin-5 (IL-5) plays a central role in eosinophil maturation, recruitment, and survival, making IL-5 inhibition a potential therapeutic strategy for eosinophil-mediated disorders [6]. We present a case of a patient diagnosed with EU, following a prior diagnosis of eosinophilic ulcerative cystitis, who demonstrated symptomatic and histologic improvement after treatment with mepolizumab, an anti-IL-5 monoclonal antibody that has shown efficacy in HESs and other eosinophilic conditions [6]. While the use of IL-5 blockade in EU remains investigational, this case highlights a potential targeted therapeutic approach for persistent eosinophilic inflammation.

## Case presentation

**Figure 1:**
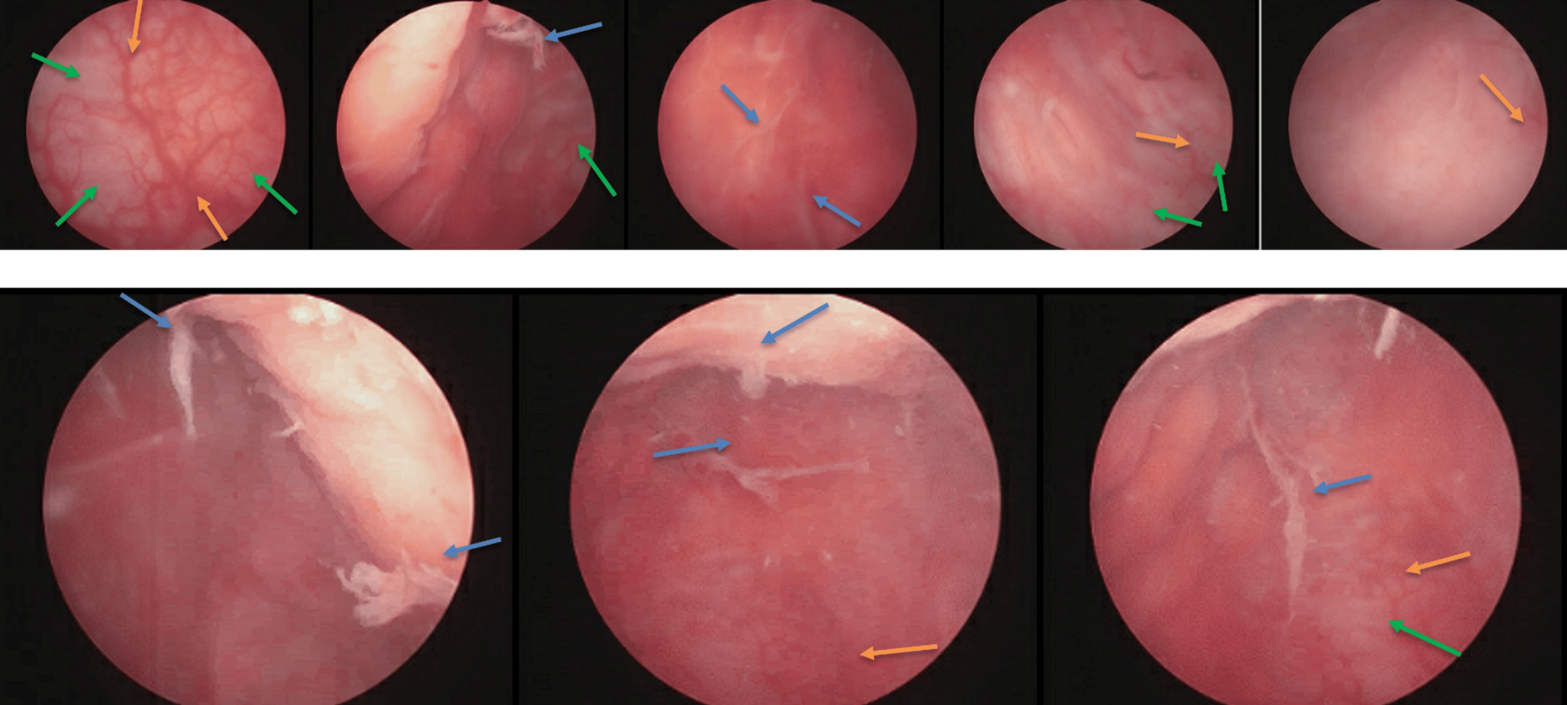
Cystoscopic imaging showing leukoplakia (blue arrow), diffuse bullous edema (green arrow), and erythema of the bladder (orange arrow).

A 54-year-old woman with a history of psoriatic arthritis, asthma, allergic rhinitis, and rosacea initially presented with increased urinary frequency, urgency, and gross hematuria. Cystoscopic evaluation revealed an abnormal bladder with diffuse bullous edema involving the right bladder wall and right vesicoureteral orifice, with vesicoureteral reflux occurring at approximately 60 mL of bladder filling. Repeat cystoscopy demonstrated progressive ulceration and inflammation involving the left and posterior bladder walls, with persistent edema on the right side. Reflux was subsequently observed at only 40 mL of bladder filling, attributed to severe transmural inflammation of the bladder (Figure 1).

**Figure 2:**
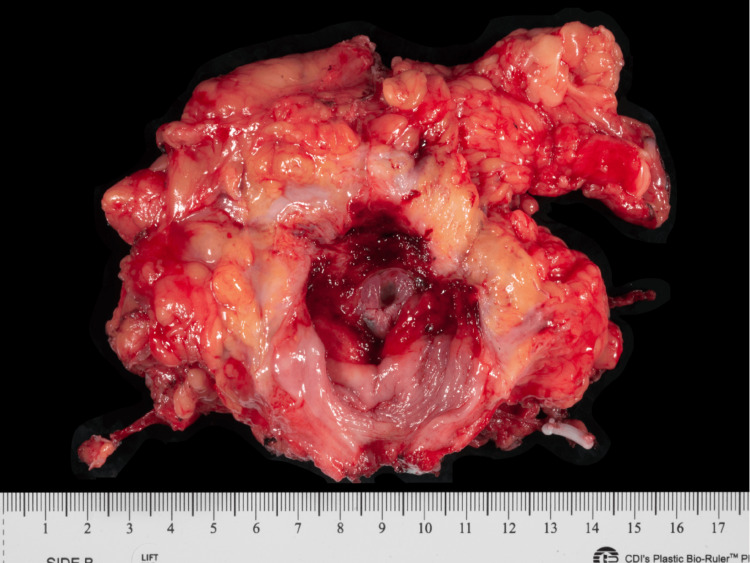
Gross pathological specimen of the bladder following cystectomy. The specimen demonstrates diffuse mucosal ulceration and inflammatory changes involving the bladder wall. Histopathologic examination of bladder biopsies obtained prior to cystectomy demonstrated dense eosinophilic infiltration, confirming the diagnosis of eosinophilic ulcerative cystitis.

A bladder biopsy performed in April 2020 confirmed a diagnosis of eosinophilic ulcerative cystitis. Due to worsening pain, gross hematuria, and end-stage bladder dysfunction, the patient underwent a cystectomy with ileal conduit diversion in January 2021. The gross pathology of the excised bladder is shown in Figure 2.

**Figure 3:**
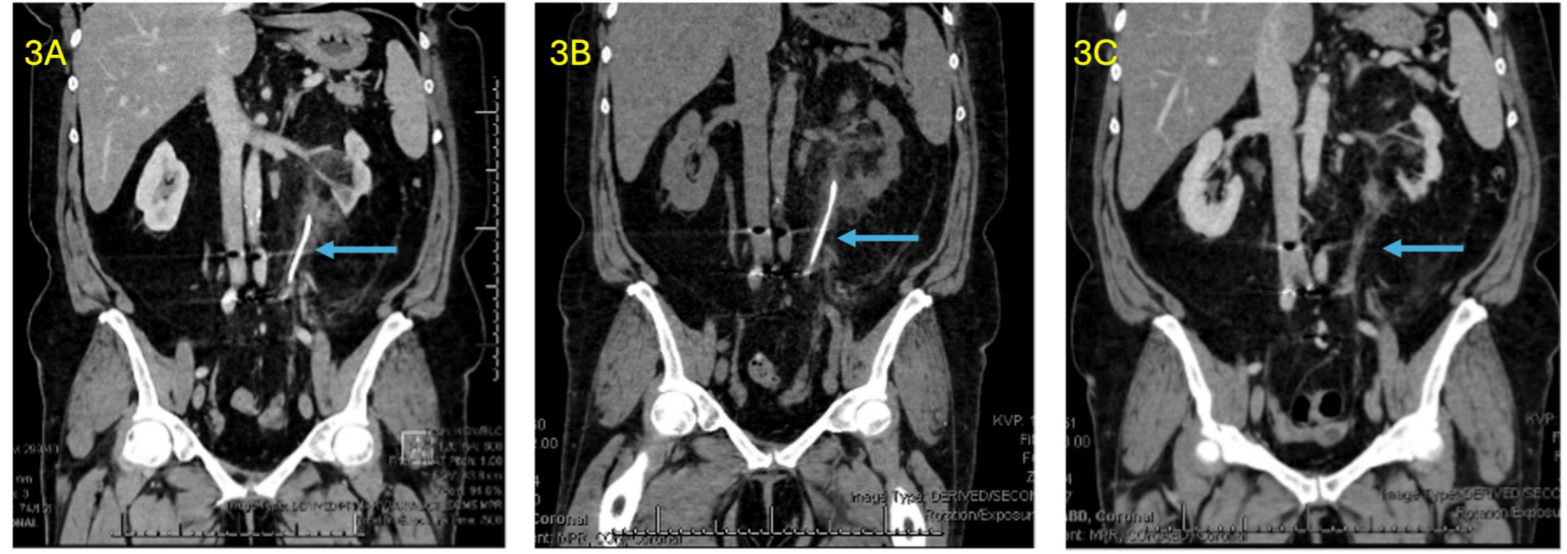
CT abdomen/pelvis with/without contrast. (3A) Post-procedural imaging following interventional radiology placement of a percutaneous nephrostomy tube and antegrade ureteral stent for treatment of left-sided hydronephrosis and ureteral obstruction. (3B) Imaging obtained after completion of a six-week course of systemic corticosteroids demonstrating partial improvement in ureteral inflammation. (3C) Follow-up imaging after initiation of mepolizumab therapy demonstrating further improvement in ureteral inflammatory changes and resolution of hydronephrosis. CT: computed tomography.

Approximately one year following cystectomy, the patient developed progressive left flank pain, and imaging demonstrated left hydronephrosis. Computed tomography (CT) of the abdomen and pelvis demonstrated inflammation and increased enhancement extending from the left renal pelvis through the ureter to the ureteral-ileal conduit anastomosis, without a discrete obstructing lesion. In the setting of hydronephrosis and suspected inflammatory ureteral involvement, interventional radiology performed percutaneous nephrostomy tube placement with left antegrade ureteral stent placement to decompress the collecting system and maintain ureteral patency. Post-procedural imaging following stent placement is shown in Figure 3A. Both the nephrostomy tube and ureteral stent were subsequently removed without complication.

Due to persistent flank pain following nephrostomy tube and ureteral stent removal, the patient underwent ureteroscopic evaluation with biopsy of the left ureter and renal pelvis later in 2021. Histopathologic examination demonstrated urothelial mucosa with acute and chronic inflammation and scattered eosinophils, establishing the diagnosis of EU (Figure 5A).

Based on biopsy-confirmed eosinophilic inflammation and prior reports demonstrating responsiveness of eosinophilic urinary tract disorders to corticosteroids, the patient was initiated on systemic corticosteroid therapy. After completion of a six-week course of corticosteroids, repeat ureteroscopy with biopsy was performed. Imaging at that time demonstrated partial radiographic improvement of ureteral inflammation (Figure 3B); however, repeat histopathology continued to demonstrate eosinophilic infiltration, consistent with persistent EU (Figure 5B).

Given the patient’s history of severe asthma and psoriatic arthritis, rheumatology was consulted regarding further immunomodulatory therapy. The patient had previously received several therapies for psoriatic arthritis, including apremilast (Otezla), methotrexate, adalimumab (Humira), ustekinumab (Stelara), and secukinumab (Cosentyx). At the time biologic therapy was considered, she remained on apremilast and low-dose methotrexate, which were continued for ongoing management of psoriatic arthritis.

Following persistent EU after corticosteroid therapy, the patient was started on mepolizumab in November 2022, administered at a dose of 300 mg subcutaneously every four weeks (three 100-mg injections), primarily for the management of severe eosinophilic asthma. Because of its mechanism of IL-5 inhibition, this therapy was also considered biologically plausible for reducing eosinophil-mediated inflammation in EU.

**Figure 4:**
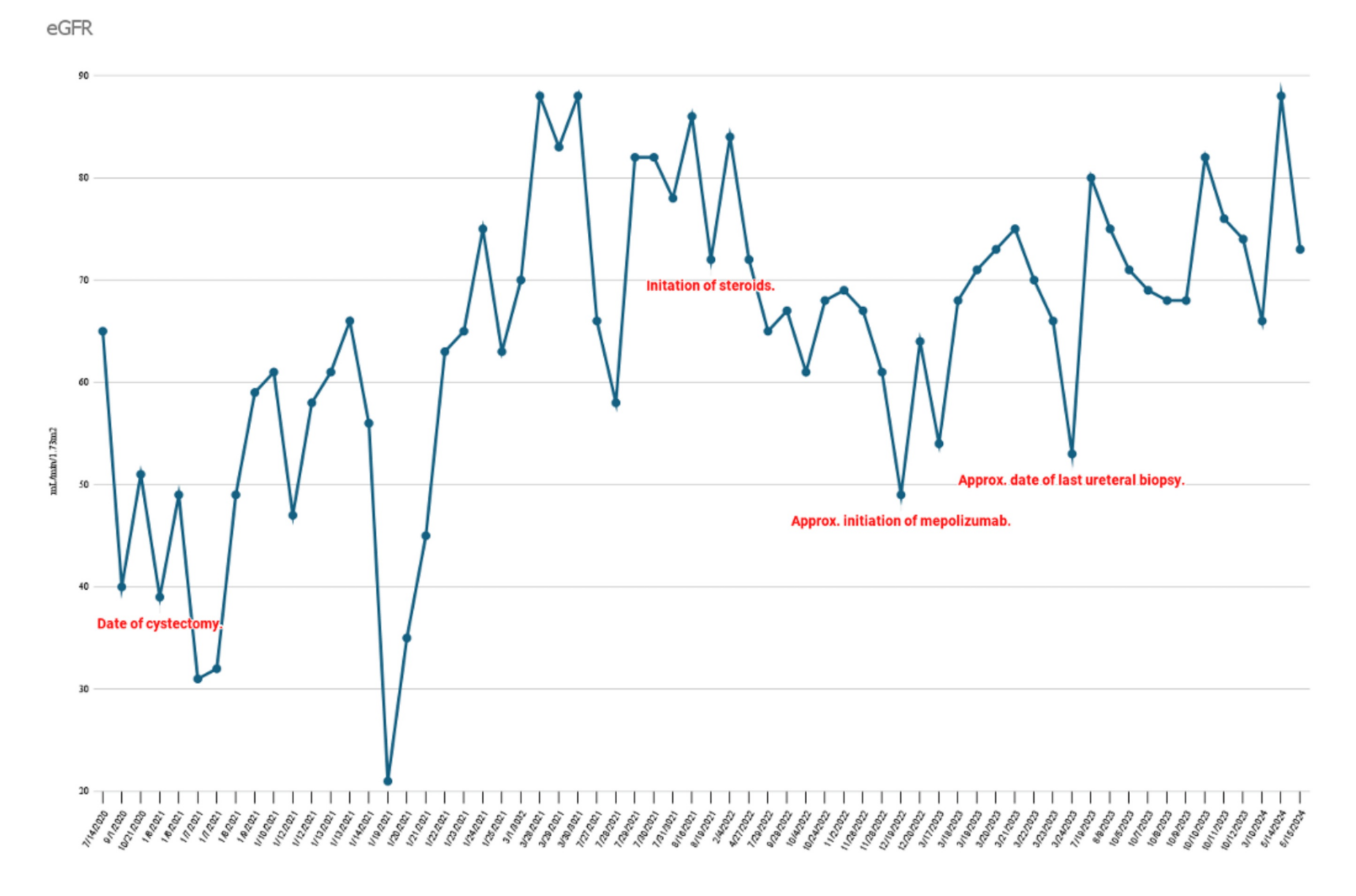
Longitudinal trend in the patient’s estimated glomerular filtration rate (eGFR) from July 14, 2020, through the most recent follow-up. Key clinical events are annotated, including cystectomy, initiation of corticosteroid therapy, initiation of mepolizumab, and the timing of repeat ureteral biopsy. Renal function demonstrates stabilization following treatment of eosinophilic ureteral inflammation.

Laboratory monitoring throughout the patient’s clinical course demonstrated fluctuations in renal function corresponding with periods of ureteral inflammation and treatment. Estimated glomerular filtration rate (eGFR) values ranged from approximately 30 to 65 mL/min/1.73 m² during the earlier stages of disease, with a nadir occurring around the time of cystectomy (Figure 4). Following treatment of ureteral inflammation, renal function gradually improved. After initiation of corticosteroids and subsequent biologic therapy, most eGFR measurements ranged between 70 and 90 mL/min/1.73 m², indicating stabilization of renal function (Figure 4).

Notably, despite biopsy-confirmed eosinophilic inflammation within the ureter, peripheral blood eosinophil counts remained within normal limits throughout the patient’s clinical course, suggesting localized eosinophilic inflammation without systemic eosinophilia.

**Figure 5:**
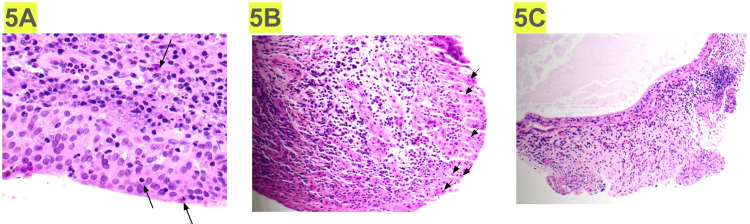
Histology. (5A) Initial ureteral biopsy demonstrating urothelial mucosa with acute and chronic inflammation and scattered eosinophils, establishing the diagnosis of eosinophilic ureteritis. (5B) A repeat biopsy obtained following corticosteroid therapy demonstrating persistent eosinophilic infiltration, indicating an incomplete response to steroid treatment. (5C) A follow-up biopsy after initiation of mepolizumab therapy demonstrating resolution of eosinophilic infiltration with residual chronic inflammatory changes.

Approximately three months after initiation of mepolizumab, the patient reported substantial improvement in flank pain. Follow-up ureteroscopic biopsies performed several months after initiation of therapy demonstrated a complete absence of eosinophils, with histology showing only chronic inflammatory changes (Figure 5C). In addition, repeat CT imaging demonstrated improvement in ureteral inflammation (Figure 3C).

At the time of the most recent follow-up, approximately 18 months after initiation of mepolizumab therapy, the patient remained clinically stable with sustained symptom control. She no longer required ureteral stents or nephrostomy tubes and reported complete resolution of flank pain.

## Discussion

EU is a rare inflammatory disorder characterized by eosinophilic infiltration of the ureteral wall, often resulting in ureteral obstruction and hydronephrosis. Due to its rarity, the pathogenesis of EU remains incompletely understood, and treatment strategies are largely derived from isolated case reports and small case series [1-3]. In most reported cases, management has included corticosteroid therapy, ureteral stenting, or surgical resection of the affected ureteral segment when obstruction persists [1-3].

The present case is notable for the involvement of multiple segments of the urinary tract, beginning with eosinophilic ulcerative cystitis and later progressing to EU. Although eosinophilic cystitis has been associated with other eosinophilic inflammatory disorders, progression of disease within the urinary tract from the bladder to the ureter has rarely been described. This case, therefore, contributes to the limited literature describing eosinophilic inflammatory disease affecting multiple components of the urinary tract.

The underlying mechanisms responsible for eosinophilic inflammation within the urinary tract are not fully elucidated but are thought to involve immune-mediated processes similar to those described in eosinophilic cystitis. Proposed triggers include epithelial injury, allergens, microbial antigens, or immune dysregulation, which may initiate an inflammatory cascade leading to eosinophil recruitment and tissue infiltration [7]. A key mediator of this process is IL-5, which plays an important role in eosinophil maturation, activation, and survival within tissues. Through downstream signaling pathways, including the JAK-STAT cascade, IL-5 promotes eosinophil proliferation and prolongs eosinophil survival at sites of inflammation [7]. Persistent activation of these pathways may contribute to chronic eosinophilic infiltration of urothelial tissue and may explain the progressive inflammatory changes observed in this patient.

Mepolizumab is a humanized monoclonal antibody of the IgG1 κ subtype that targets IL-5, preventing its interaction with the α-chain of the IL-5 receptor and thereby reducing eosinophil activation and survival [6,8]. The drug is currently FDA-approved for the treatment of severe eosinophilic asthma, eosinophilic granulomatosis with polyangiitis (EGPA), and HES [6,9]. Because IL-5 signaling plays a central role in eosinophil-mediated inflammation, inhibition of this pathway represents a biologically plausible therapeutic strategy for conditions characterized by eosinophilic tissue infiltration.

In this case, the decision to initiate anti-IL-5 pathway therapy was made collaboratively between urology and rheumatology after persistent biopsy-proven eosinophilic inflammation of the urinary tract despite corticosteroid therapy and multiple procedural interventions. The dosing strategy followed the regimen used for EGPA and HES, consisting of 300 mg administered as three 100-mg subcutaneous injections every four weeks rather than the lower dosing used for severe eosinophilic asthma [9]. This approach was selected given the refractory nature of the patient’s disease and the involvement of multiple segments of the urinary tract.

Other IL-5 pathway inhibitors were considered but were less suitable in this context. Reslizumab requires intravenous administration and carries a boxed warning for anaphylaxis, whereas mepolizumab is administered subcutaneously and has an established safety profile that facilitates outpatient treatment [6]. Benralizumab targets the IL-5 receptor and induces antibody-dependent cellular cytotoxic depletion of eosinophils; however, at the time of treatment initiation, mepolizumab had a longer clinical track record in systemic eosinophilic diseases [6,9].

An additional notable feature of this case was the absence of peripheral eosinophilia despite significant eosinophilic infiltration on tissue biopsy. This phenomenon has been described in other eosinophilic inflammatory disorders and highlights the importance of tissue sampling in establishing the diagnosis [7]. Localized eosinophilic inflammation may occur even when circulating eosinophil levels remain within normal limits, suggesting that peripheral eosinophil counts alone may not reliably reflect disease activity in tissue-restricted eosinophilic conditions.

Following initiation of mepolizumab therapy, the patient experienced significant clinical improvement with resolution of flank pain and eventual removal of ureteral stents and percutaneous nephrostomy tubes. Repeat biopsies demonstrated remission of active eosinophilic inflammation, with histologic findings showing chronic inflammatory changes without persistent eosinophilic infiltration. Although the temporal association between initiation of mepolizumab and disease improvement is notable, a causal relationship cannot be definitively established based on a single case.

Several limitations should be considered when interpreting these findings. This report describes a single patient and therefore cannot establish the efficacy of IL-5-targeted therapy for EU. Additionally, the patient received multiple prior treatments, including corticosteroids and other immunomodulatory therapies, which may confound the interpretation of the observed clinical response. Spontaneous disease fluctuation or delayed effects of prior therapies also cannot be excluded. Nevertheless, the observed clinical and histologic improvement following IL-5 inhibition suggests that biologic therapy targeting eosinophilic pathways may warrant further investigation in refractory eosinophilic urinary tract disease.

Given the rarity of EU and the absence of standardized treatment protocols, this case highlights the importance of multidisciplinary collaboration in the management of complex eosinophilic inflammatory disorders. Additional reports and further investigation will be necessary to better characterize the pathophysiology of EU and determine whether targeted biologic therapies may represent a viable treatment strategy for patients with severe or refractory disease.

## Conclusions

We describe the use of the IL-5 monoclonal antibody mepolizumab in a patient with EU diagnosed following radical cystectomy for end-stage eosinophilic cystitis who had been refractory to corticosteroid management. Both clinically and histologically, the patient’s condition improved following treatment with this biologic agent. This case highlights potential aspects of disease pathogenesis and expands consideration of targeted biologic therapies for eosinophilic inflammatory disorders of the urinary tract. However, given that this report describes a single patient, these findings should be interpreted with caution and should not yet guide standard clinical management. Further investigation through additional case series, prospective studies, and rare disease registries will be necessary to better understand the pathogenesis of EU and define the potential role of IL-5-targeted therapies in its management.
